# A novel application of the submucosal tunnel technique for resection of a giant duodenal lipoma

**DOI:** 10.1055/a-2760-9072

**Published:** 2026-01-22

**Authors:** Jingjing Lian, Aiping Xu, Tao Chen, Meidong Xu

**Affiliations:** 1 66324 Endoscopy Center, Department of Gastroenterology, Shanghai East Hospital, Tongji University School of Medicine, Shanghai, China

**Fig. 1:**
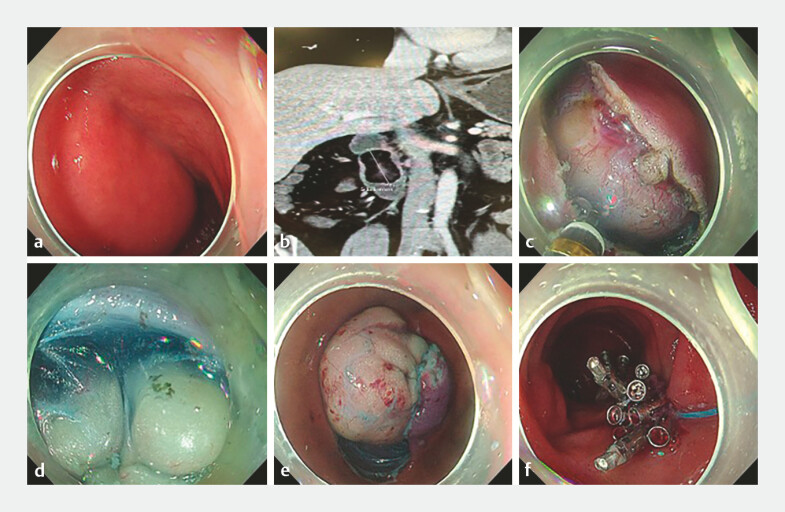
Endoscopic resection of a giant duodenal lipoma using a submucosal tunnel technique. **a** Endoscopic view of the large, submucosal mass in the descending duodenum. **b** Axial abdominal MRI findings of the duodenal lipoma. **c** A horizontal mucosal incision was made at the proximal edge of the tumor. **d** Submucosal dissection was performed, creating a tunnel above the yellow fatty tissue of the lipoma. **e** The tumor was gradually extruded into the lumen. **f** The mucosal entry site was closed with endoclips and secured with a nylon loop.

A 47-year-old male was admitted for investigation of melena. Esophagogastroduodenoscopy revealed a large, smooth, submucosal mass in the descending duodenum ( Fig. 1 **a** ). The patient underwent endoscopic ultrasound and abdominal magnetic resonance imaging (MRI) for further characterization of the duodenal mass. Both studies were unequivocally consistent with a submucosal lipoma ( Fig. 1 **b** ). The lesion was estimated to be >5 cm in the greatest diameter.

Given the symptomatic nature of the lesion, endoscopic resection was indicated. Prior to dissection, we introduced a side-viewing duodenoscope to repeatedly verify that the lesion was unrelated to the papilla.

**Fig. 2:**
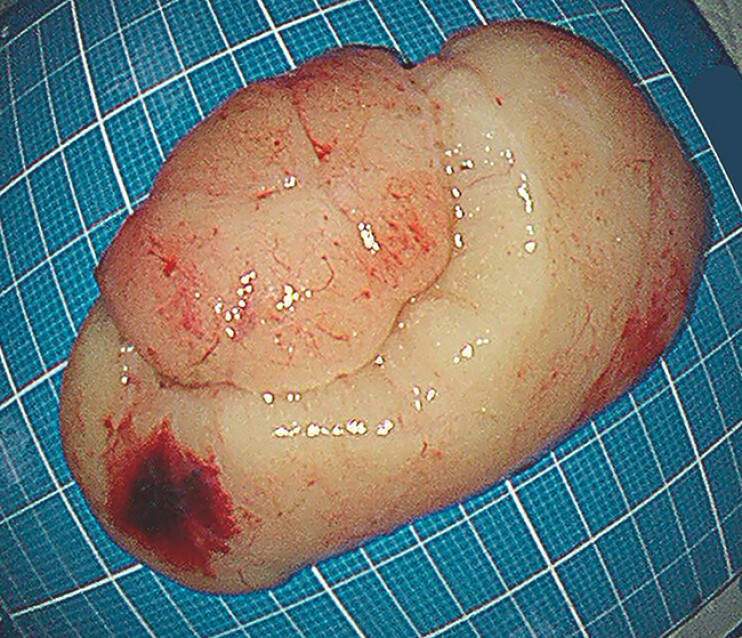
The resected lipoma specimen measuring 7.0 cm × 4.0 cm.

However, due to its enormous size and broad base, a standard ESD was deemed high-risk, as the resulting defect would be massive and impossible to close securely, posing a significant risk of perforation. We here presented a novel application of submucosal tunnel resection, allowing for en-bloc removal while preserving the overlying mucosa. The steps were as follows ( Video 1 , Fig. 1 ): First, a 3-cm horizontal mucosal incision was made at the oral (proximal) edge of the tumor following submucosal injection ( Fig. 1 **c** ). Then, the submucosal layer was dissected carefully above the tumor capsule to create a tunnel and the dissection was advanced distally between the mucosal layer and the tumor mass ( Fig. 1 **d** ). As dissection proceeded, the tumor was gradually extruded into the lumen ( Fig. 1 **e** ). Next, the final connection was cut, and the tumor was immediately captured and retrieved en-bloc with a snare. Finally, the mucosal incision site and the small residual defect were closed with endoclips and finally secured with a nylon loop ( Fig. 1 **f** ). The resected specimen measured 7.0 cm × 4.0 cm ( Fig. 2 ).

The process of endoscopic resection of a giant duodenal lipoma.Video 1

The patient recovered uneventfully, was started on a clear liquid diet on postoperative day 2, and was discharged on day 4. Pathological examination confirmed the diagnosis of a lipoma.

To our knowledge, this is the first report of a submucosal tunnel technique being used for a large duodenal lesion. It represents a valuable addition to the therapeutic endoscopist's arsenal for managing complex SMTs in this challenging anatomical location.

Endoscopy_UCTN_Code_TTT_1AO_2AG_3AZ

